# Identification and validation of a prognostic anoikis-related gene signature in papillary thyroid carcinoma by integrated analysis of single-cell and bulk RNA-sequencing

**DOI:** 10.1097/MD.0000000000038144

**Published:** 2024-05-10

**Authors:** Ke Zheng, Xiu-Xia Zhang, Xin Yu, Bin Yu, Yi-Fei Yang

**Affiliations:** a Department of Thyroid and Breast Surgery, Linping Campus, The Second Affiliated Hospital of Zhejiang University School of Medicine, Hangzhou, China.

**Keywords:** anoikis, drug sensitivity, immune escape, papillary thyroid carcinoma, single-cell RNA

## Abstract

Papillary thyroid carcinoma (PTC) prognosis may be deteriorated due to the metastases, and anoikis palys an essential role in the tumor metastasis. However, the potential effect of anoikis-related genes on the prognosis of PTC was unclear. The mRNA and clinical information were obtained from the cancer genome atlas database. Hub genes were identified and risk model was constructed using Cox regression analysis. Kaplan–Meier (K–M) curve was applied for the survival analysis. Immune infiltration and immune therapy response were calculated using CIBERSORT and TIDE. The identification of cell types and cell interaction was performed by Seurat, SingleR and CellChat packages. GO, KEGG, and GSVA were applied for the enrichment analysis. Protein-protein interaction network was constructed in STRING and Cytoscape. Drug sensitivity was assessed in GSCA. Based on bulk RNA data, we identified 4 anoikis-related risk signatures, which were oncogenes, and constructed a risk model. The enrichment analysis found high risk group was enriched in some immune-related pathways. High risk group had higher infiltration of Tregs, higher TIDE score and lower levels of monocytes and CD8 T cells. Based on scRNA data, we found that 4 hub genes were mainly expressed in monocytes and macrophages, and they interacted with T cells. Hub genes were significantly related to immune escape-related genes. Drug sensitivity analysis suggested that cyclin dependent kinase inhibitor 2A may be a better chemotherapy target. We constructed a risk model which could effectively and steadily predict the prognosis of PTC. We inferred that the immune escape may be involved in the development of PTC.

## 1. Introduction

Thyroid cancer (THCA) is the most common endocrine cancer with a rapidly increasing incidence over several decades,^[1]^ and papillary thyroid carcinoma (PTC) is the main type of THCA, comprising approximately 80% to 85% of all THCA.^[2]^ While the 5-year survival rate in its incipient stages stands commendably over 90%, patients afflicted with advanced PTC encounter a considerably bleaker prognosis, with a 5-year survival rate plummeting below 60% due to the ominous convergence of metastasis and uninhibited tumor proliferation,^[3,4]^. The current treatment options for PTC include surgical resection, thyroid hormone suppression, and radioiodine therapy, all of which may result in a relatively good prognosis for patients with PTC.^[5]^ Regrettably, the clinical trajectory of PTC sufferers remains stifled by the persistent specters of recurrence and metastatic dissemination, both of which serve as primary instigators of mortality in this context.^[6]^ Thus, a comprehensive comprehension pertaining to the intricate mechanisms governing the initiation and advancement of PTC stands poised to contribute significantly to the diagnostic and therapeutic endeavors undertaken within the realm of this affliction.

Anoikis is a member of programmed cell death triggered by cell detachment from the extracellular matrix.^[7]^ This vital process assumes a paramount role as the primary line of defense against metastasis, while simultaneously orchestrating tissue homeostasis and development.^[8,9]^ Additionally, it has been extensively elucidated that anoikis protects against metastasis and aberrant proliferation of detached tumor cells, thus emerging as a pivotal player in the genesis and progression of neoplastic formations.^[10,11]^ Moreover, as the frontiers of omics technology and bioinformatics continue to expand, mounting evidence underscores the stringent influence exerted by a subset of anoikis-related genes (ARGs) upon the intricate machinery governing tumor metastasis. Windham et al identified that SRC could enhance the anoikis-resistant to promote the metastasis of tumor cells.^[12]^ Moreover, it was widely reported that ARGs could predict the prognosis of some tumors including gastric cancer, breast cancer and lung cancer.^[13–15]^ Anoikis resistance is the inherent characteristics of tumor cell,^[16]^ but there were few reports about the function of anoikis in the PTC development.

Therefore, we tried to investigate the prognosis value of ARGs in PTC and constructed a risk model based on ARGs. Besides, we also preliminarily explored the potential mechanism of anoikis in PTC development.

## 2. Methods

### 2.1. Data collection and process

The mRNA expression, clinical data and survival data (overall survival [OS] status and OS time) were downloaded from the cancer genome atlas (TCGA) database. The raw microarray mRNA data were subjected to log2 transformation, with genes having multiple probes being averaged. Besides, the single-cell RNA (scRNA) data (GSE193581) was obtained from the GEO database, and 4 PTC samples were selected for the analysis. The ARGs were determined in GeneCard (https://www.genecards.org/), and immune escape-related genes were obtained from a previous study.^[17]^

### 2.2. The identification of hub genes

The limma package in R was utilized to identify differentially expressed genes (DEGs). Besides, the threshold values for identifying DEGs were set at fold change > 1.5 and *P* < .05.

### 2.3. The construction and validation of the risk model

After the DEGs were determined, univariate Cox regression analysis was conducted to determine the genes closely related to the prognosis of PTC. Then, the least absolute shrinkage and selection operator regression analysis was used to prevent over-fitting using the glmnet package. Next, the hub genes and corresponding coefficients were determined using multivariate Cox analysis, and the risk model was constructed based on the expression and coefficient value of hub genes. Additionally, the patients were divided into low and high risk groups based on the optimal cutoff value using the maxstat package. Finally, the Kaplan–Meier (K–M) and operating characteristic curve (ROC) curves were performed to validate the stability and suitability of the risk model using survival and qROC packages.

### 2.4. The immune infiltration and immune response analysis

The CIBERSORT algorithm, a deconvolution tool, was utilized to calculate the abundance of 22 immune cells using the CIBERSORT package. Tumor Immune Dysfunction and Exclusion (TIDE) is a computational approach designed to forecast the effectiveness of immune checkpoint blockade therapy in cancer patients, which was a more reliable biomarker than TMB or PD-L1 expression in determining the response to immunotherapy.^[18]^ After the raw expression profiles were normalized using z-scores, TIDE, microsatellite instability (MSI), Dysfunction and Exclusion scores were calculated to predict the immunotherapy response in the TIDE website (http://tide.dfci.harvard.edu/). A lower TIDE score suggests a reduced likelihood of immune escape.

### 2.5. The single-cell RNA analysis

Before the analysis of scRNA data, the cells with the number of nFeature RNA <500 were excluded, and the cells with the number of nCount RNA <1000 or more than 20,000 were filtered out, and the cells with mitochondria percent more than 10% were excluded. Overall, a total of 44,842 cells were chosen for further analysis. After the determination of the optimal dimension using principal component analysis, t-SNE was used for dimensionality reduction and cluster identification. Then, we identify marker genes through the Find All Markers function with min. pct = 0.25 and log2 (Fold change) = 0.3. Finally, the SingleR package was used for the cluster annotation. Then, the cell interaction and the interactions of ligand and receptor were estimated using the Cell Chat package.

### 2.6. The enrichment analysis

The ClusterProfiler package was performed for Gene Ontology (GO) and Kyoto Encyclopedia of Genes and Genomes (KEGG) analysis. Gene set variation analysis (GSVA) package was used for GSVA analysis of the differential marker genes between 2 groups. All reference gene sets were obtained in the Molecular Signatures Database (MSigDB, https://www.gsea-msigdb.org/gsea/msigdb/index.jsp).

### 2.7. Cell culture and quantitative real-time polymerase chain reaction (qRT-PCR)

The normal thyroid cells (Htori-3) and THCA cells (TPC-1) were obtained from the American Type Culture Collection (American Type Culture Collection, Manassas, VA, USA), maintained in DEME/F12 medium (Hyclone, UT, USA) containing 10% fetal bovine serum (FBS) and 1% penicillin/streptomycin and incubated in 5% CO_2_ at 37°C.

Total RNA was extracted from cells using Trizol Reagent (15596026, Invitrogen, Car, CA, USA). The Nanodrop 2000 Micro-UV Spectrophotometer (1011U, NanoDrop Technologies, Wilmington, DE, USA) was employed to determine the ratio of A260/A230 to A260/A280. After RNA samples were measured, they were reversely transcribed into cDNA with PrimeScript™ RT reagent Kit (RR037A, Takara Bio Inc., Osaka, Japan). qRT-PCR was conducted using TB Green qPCR Master Mix (639676, Takara Biotechnology, Japan) in the Mx3005P system (Agilent Technologies, Inc., CA, USA). The expressions of hub genes were determined using the 2^-ΔΔCt^ method with glyceraldehyde-3-phosphate dehydrogenase as control (32). PCR reaction conditions were: 1 minute at 95°C, then 20 seconds at 95°C, and 45 seconds at 58°C for 40 cycles. The primer sequences are shown in Table 1.

**Table 1:** All primers in qRT-PCR experiments in this study.

Gene	Forward	Reverse
CDKN2A	5’-CCACGGCGCGGAGCCCAA-3’	5’-GCAGCACCACCAGCGTGTCCA-3’
IRX1	5’-CCTATGGTCAGTTTCAATACG-3’	5’-GGTCATCTTGGTGATAATGG-3’
SCD1	5’-CTGGCCTATGACCGGAAGAAA-3’	5’-GACCCCAAACTCATTCCATAGG-3’
SNAI1	5’-CTCGGACCTTCTCCCGAATG-3’	5’-AAAGTCCTGTGGGGCTGATG-3’

CDKN2A = cyclin dependent kinase inhibitor 2A, IRX1 = Iroquois homeobox 1, SCD1 = stearoyl-CoA desaturase 1, SNAI1 = Snail family transcriptional repressor 1.

### 2.8. The construction of protein-protein interaction network

After the hub gene list was uploaded to STRING (https://cn.string-db.org/), the interaction among proteins was assessed. Then, the protein-protein interaction (PPI) network was analyzed, adjusted and visualized in Cytoscape.

### 2.9. The analysis of drug sensitivity

Gene Set Cancer Analysis (GSCA) is a comprehensive platform integrating genomic, pharmacogenomic and immunogenomic GSCA. Besides, GSCA incorporates over 750 small-molecule drugs from the Genomics of Drug Sensitivity in Cancer (GDSC) and the Cancer Therapeutics Response Portal (CTRP) databases.

### 2.10. Statistics

All statistical analyses were performed using R software v4.3.1. The log-rank test was used to assess the statistical difference between the 2 groups. Spearman correlation analysis was performed for the correlation analysis. *P*-values < .05 were considered statistically significant.

## 3. Results

### 3.1. Identification of differentially expressed anoikis-related genes

**Figure 1.**
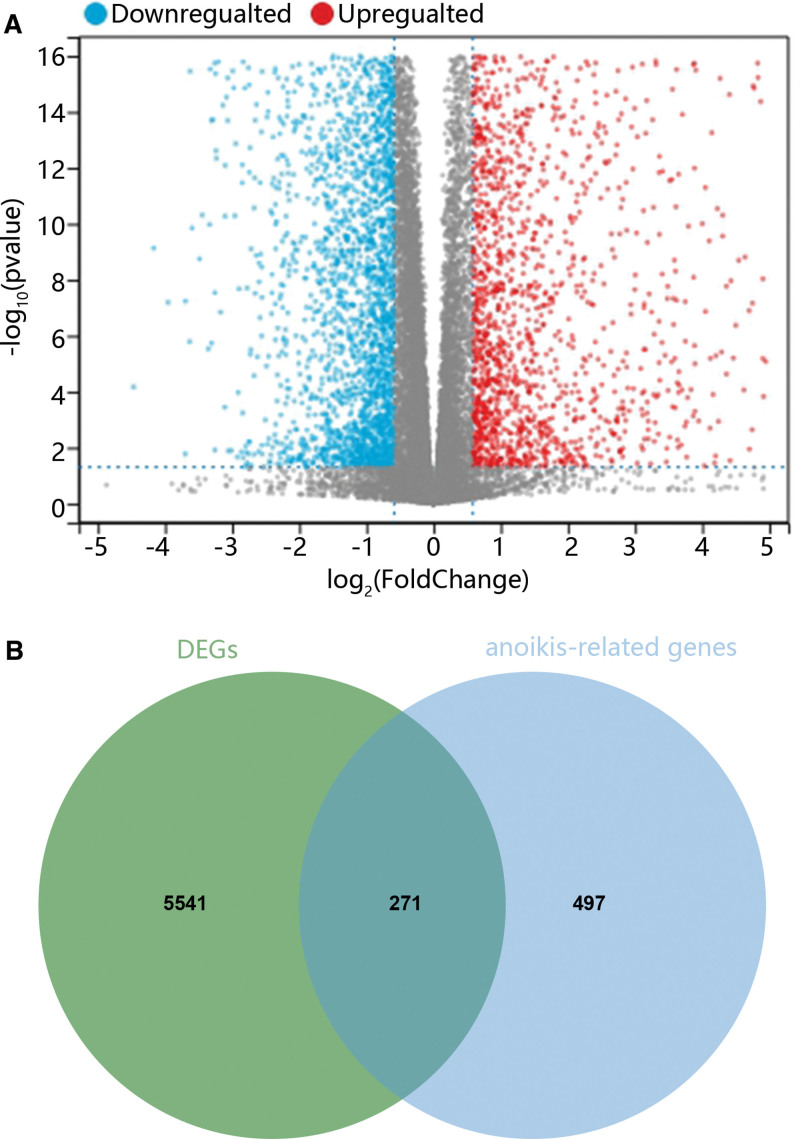
The selection of DEARGs. (A) The genes differentially expressed in normal and PTC groups. (B) Venn plots showed the overlapping genes. DEARGs = differentially expressed ARGs, PTC = papillary thyroid carcinoma.

At first, we analyze gene expression differences using bioinformatics. As shown in Figure 1A, there were 2971 upregulated genes and 2841 downregulated genes in PTC samples compared with normal samples. Combined with the dataset of ARGs, we found that 271 ARGs were differentially expressed between tumor and normal samples (Fig. 1B).

### 3.2. Construction and validation of risk model

**Figure 2.**
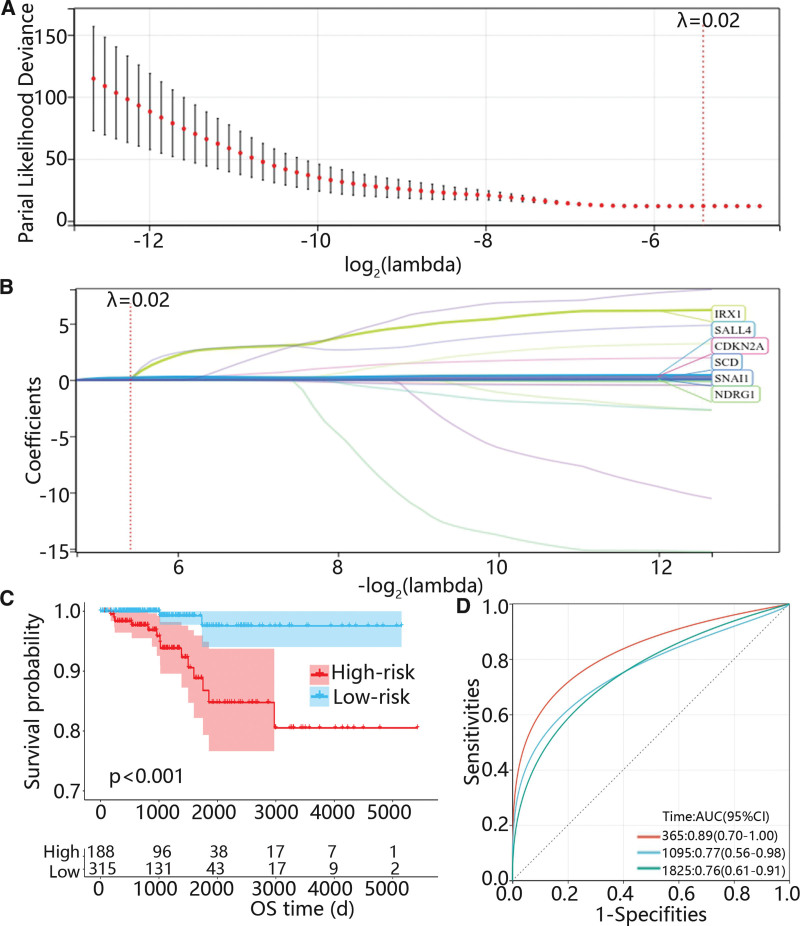
The identification and validation of anoikis-related signature in TCGA cohorts. (A–B) Lasso Cox analysis identified 6 signatures related to the prognosis of PTC. (C) K–M curve of risk model based on TCGA database. (D) ROC curve of risk model for 1-, 3- and 5-year prognosis of PTC. PTC = papillary thyroid carcinoma, TCGA = the cancer genome atlas.

Then, a total of 93 ARGs related to the prognosis of PTC were screened out using univariate Cox regression analysis. Besides, the Lasso Cox was used to further exclude some genes which may be highly associated with other genes, and 6 genes were selected when λ was 0.02 (Figs. 2A-B). Subsequently, multivariate Cox regression analysis was applied to determine the mRNA which could independently predict the prognosis of PTC and corresponding coefficients to construct a risk model. The risk score = 0.168 * expression (cyclin dependent kinase inhibitor 2A [CDKN2A]) + 3.296 * expression (Iroquois homeobox 1 [IRX1]) + 0.07 * expression (stearoyl-CoA desaturase [SCD]) + 0.107 * expression (Snail family transcriptional repressor 1 [SNAI1]). Furthermore, the patients were divided into low and high risk groups according to the optimal cutoff, and the K–M curve displayed that the overall survival probability of the high-risk group was significantly lower than that of the low-risk group (Fig. 2C). Moreover, the area under the curve values for predicting 1-, 3- and 5-years survival probability were 0.89, 0.77 and 0.76, respectively (Fig. 2D).

### 3.3. The function enrichment analysis between high and low risk groups

**Figure 3.**
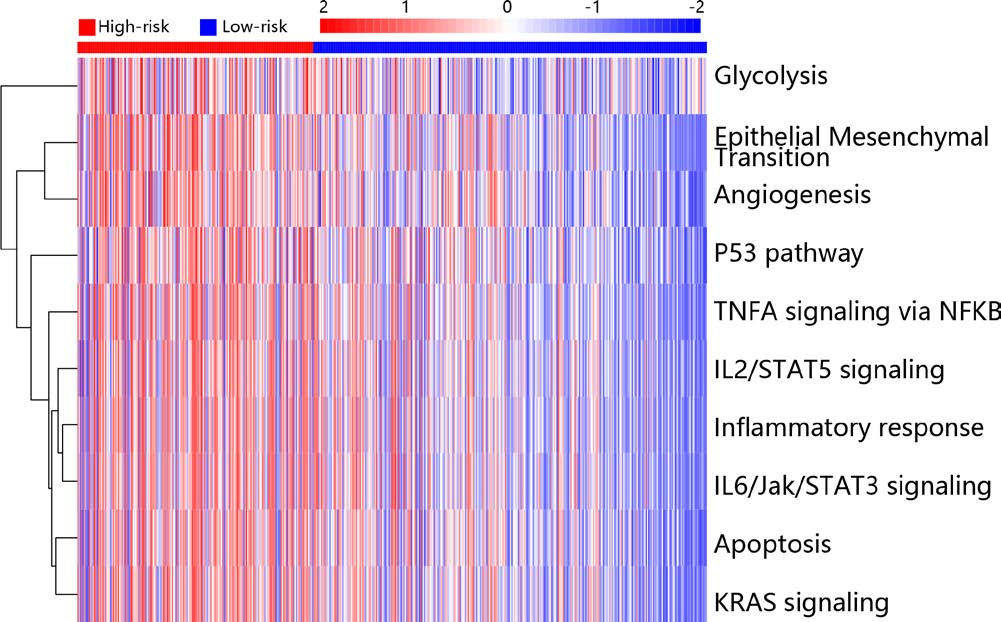
Heatmap exhibited the differential enrichment pathways between the high risk and low risk groups.

To explore the potential mechanism in the development of PTC, the GSVA analysis was used to assess the differentially enriched pathways in low and high risk groups. The top 10 pathways were shown in Figure 3, and it can be seen that glycolysis, epithelial mesenchymal transition (EMT) and angiogenesis were highly enriched in the high risk group.

### 3.4. The immune response and immune infiltration between high and low risk groups

**Figure 4.**
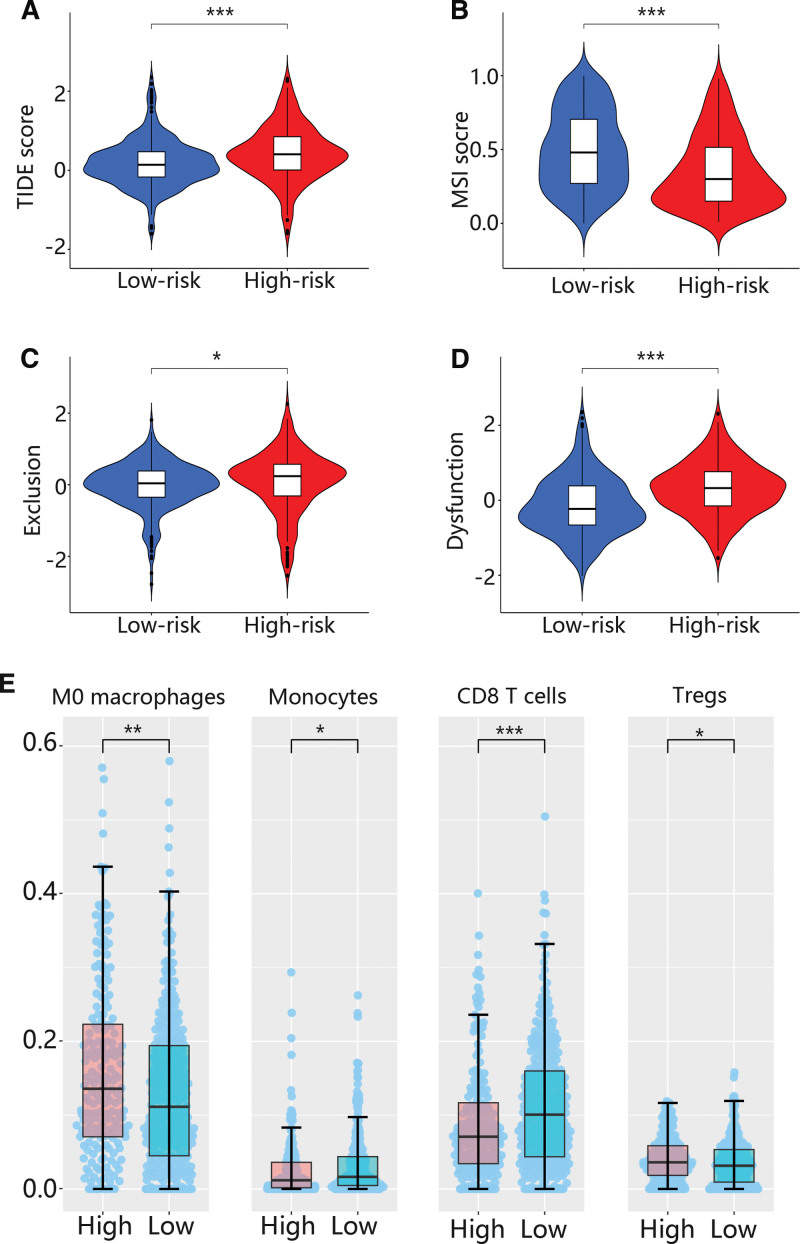
The immune response and immune infiltration were assessed by TIDE and cibersort. (A) The TIDE score in low and high risk groups. (B) The MSI score in low and high risk groups. (C) The exclusion score in low and high risk groups. (B) The dysfunction score in low and high risk groups. (B) The infiltration levels of 4 immune cells in low and high risk groups. MSI = microsatellite instability.

Because some results were related to the immune system, we further analyzed the immune response using TIDE. The TIDE score was higher in the high risk group than that of the low risk group (Fig. 4A), while MSI, exclusion and dysfunction scores were lower in the high risk group than that of the low risk group (Figs. 4B-D). Besides, the immune infiltration levels of M0 macrophage and regulatory T cells (Tregs) were higher in high risk groups in comparison with low risk group, while the immune infiltration levels of monocytes and CD8 T cells were higher in low risk groups (Fig. 4E).

### 3.5. The single-cell RNA analysis of papillary thyroid carcinoma samples

**Figure 5.**
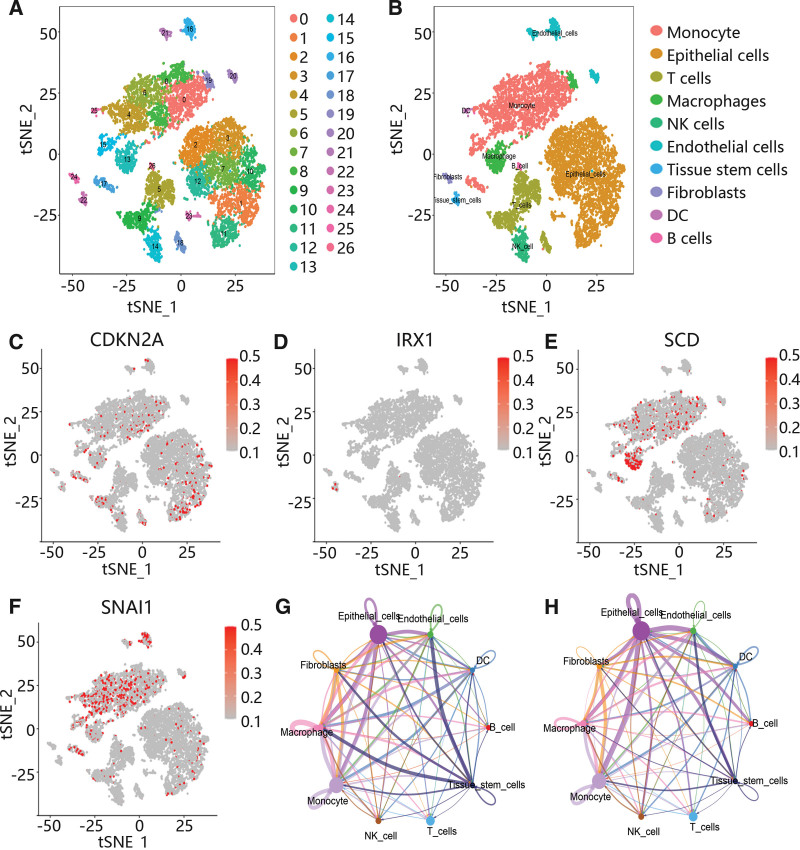
The expression of hub genes in different cell types and the interaction between various cell types. (A) Separate clusters using t-SNE. (B) Cell type annotation using SingleR. (C–F) The expression of hub genes in different cell types. (G) The number of interactions among 10 cell types. (H) The weight of interactions among 10 cell types.

Subsequently, to further explore the potential mechanism, 4 scRNA data were integrated and analyzed. After quality control and batch normalization, these cells were clustered using t-SNE. A total of 27 clusters were identified, and 10 major cell types were annotated, including monocyte, epithelial cells, T cells, macrophages, endothelial cells, tissue stem cells, fibroblasts, dendritic cell and B cells (Figs. 5A-B). Moreover, CDKN2A, SCD, and SNAI1 were highly expressed in monocytes, macrophages and epithelial cells, while IRX1 was highly expressed in tissue stem cells (Figs. 5C-F). Furthermore, the cell communication results showed that monocytes, macrophages and epithelial cells had the most and strongest interactions with other cells (Fig. 5G-H, Figure S1, Supplemental Digital Content, http://links.lww.com/MD/M495), and some receptor-ligand pairs played vital roles in the interaction among different cell types, including TGFB1/TGFbR1_R2 and TNF/ TNFRSF1B (Figure S2, Supplemental Digital Content, http://links.lww.com/MD/M496).

### 3.6. The expression and prognosis value of hub genes

**Figure 6.**
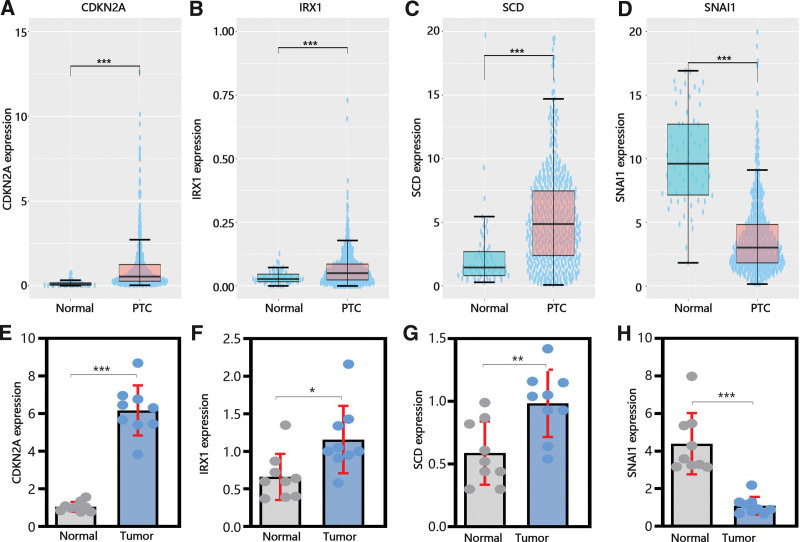
The expression of 4 hub genes. (A–D) The expression of CDKN2A, IRX1, SCD, and SNAI1 in TCGA dataset. (E–H) qRT-PCR verifying the gene transcription in tumor and normal cells. CDKN2A = cyclin dependent kinase inhibitor 2A, IRX1 = Iroquois homeobox 1, SCD = stearoyl-CoA desaturase, SNAI1 = Snail family transcriptional repressor 1, TCGA = the cancer genome atlas.

Next, to explore the function of hub genes in the development and metastasis of PTC. We determined their expression in normal and tumor samples. From Figure 6A-C, it was obvious that CDKN2A, IRX1, and SCD was upregulated in PTC samples compared with normal samples in TCGA database, while SNAI1 was downregulated in PTC samples (Fig. 6D). As similar as the results of TCGA database, qRT-PCR results also exhibited that the expression of CDKN2A, IRX1 and SCD were upregulated in PTC samples and SNAI1 was downregulated in PTC samples (Figs. 6E-H).

**Figure 7.**
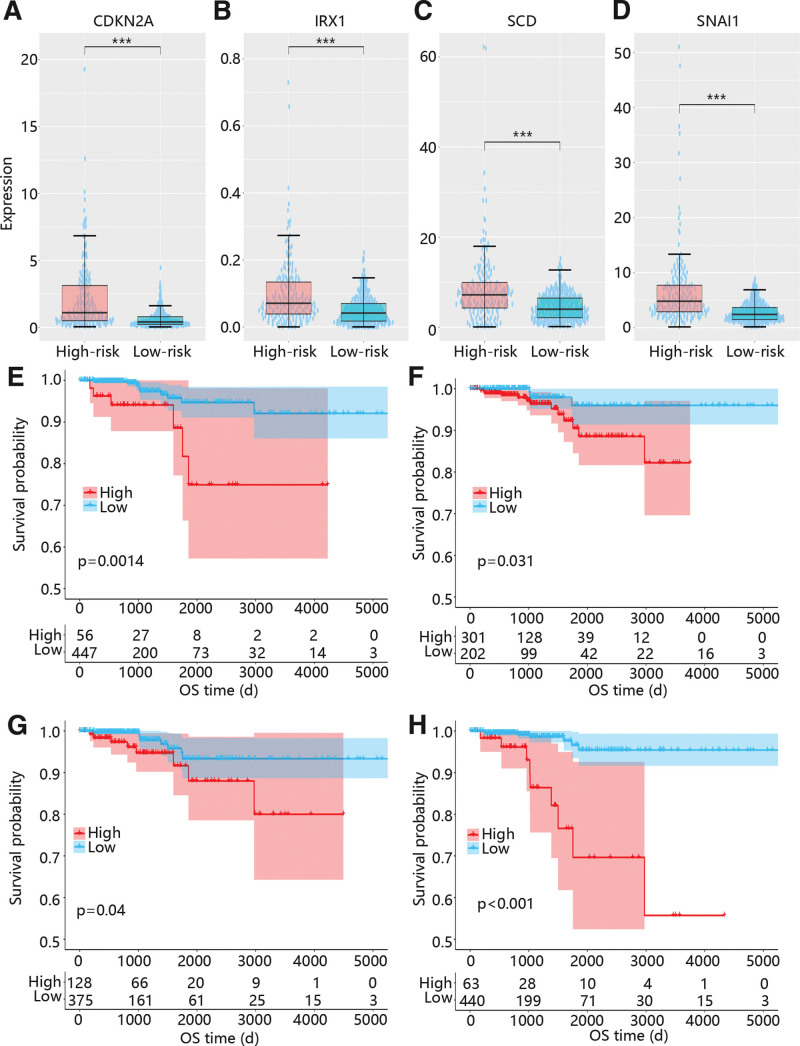
The expression of hub genes and the correlation between hub gene expressions and prognosis. (A) The CDKN2A expression in high and low risk groups. (B) The IRX1 expression in high and low risk groups. (A) The SCD expression in high and low risk groups. (A) The SNAI1 expression in high and low risk groups. (E) The K–M curve of CDKN2A in TCGA cohorts. (F) The K–M curve of IRX1 in TCGA cohorts. (G) The K–M curve of SCD in TCGA cohorts. (H) The K–M curve of SNAI1 in TCGA cohorts. CDKN2A = cyclin dependent kinase inhibitor 2A, IRX1 = Iroquois homeobox 1, SCD = stearoyl-CoA desaturase, SNAI1 = Snail family transcriptional repressor 1, TCGA = the cancer genome atlas.

As shown in Figure 7, CDKN2A, IRX1, SCD, and SNAI1 were all upregulated in high risk groups (Figs. 7A-D), and the high expressions of them were all closely related to the poor prognosis of PTC (Figs. 7E-H).

### 3.7. The correlation of hub genes with immune escape-related genes and pairs of ligand and receptor

**Figure 8.**
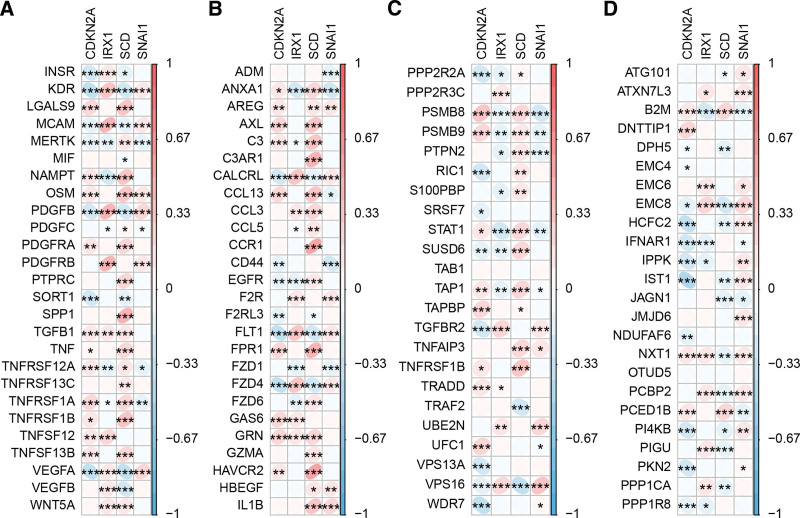
The hub genes were closely related to immune escape-related genes and pairs of ligands and receptors. (A–B) The correlation between hub genes and immune escape-related genes. (C–D) The correlation between hub genes and pairs of ligands and receptors.

Next, we analyzed the function of hub genes in the immune system. The results showed that CDKN2A, IRX1, SCD, and SNAI1 were most closely and positively related to PSMB8, VPS16, TNFRSF1B, and VPS16, respectively, based on the immune escape-related genes (Figs. 8A-B). In addition, CDKN2A, IRX1, SCD, and SNAI1 were most closely and positively related to GAS6, MCAM, CCR1, and VEGFA, respectively, based on the ligand and receptor pairs of monocytes, macrophages and epithelial cells (Figs. 8C-D).

### 3.8. The function enrichment analysis and PPI network of the hub genes

**Figure 9.**
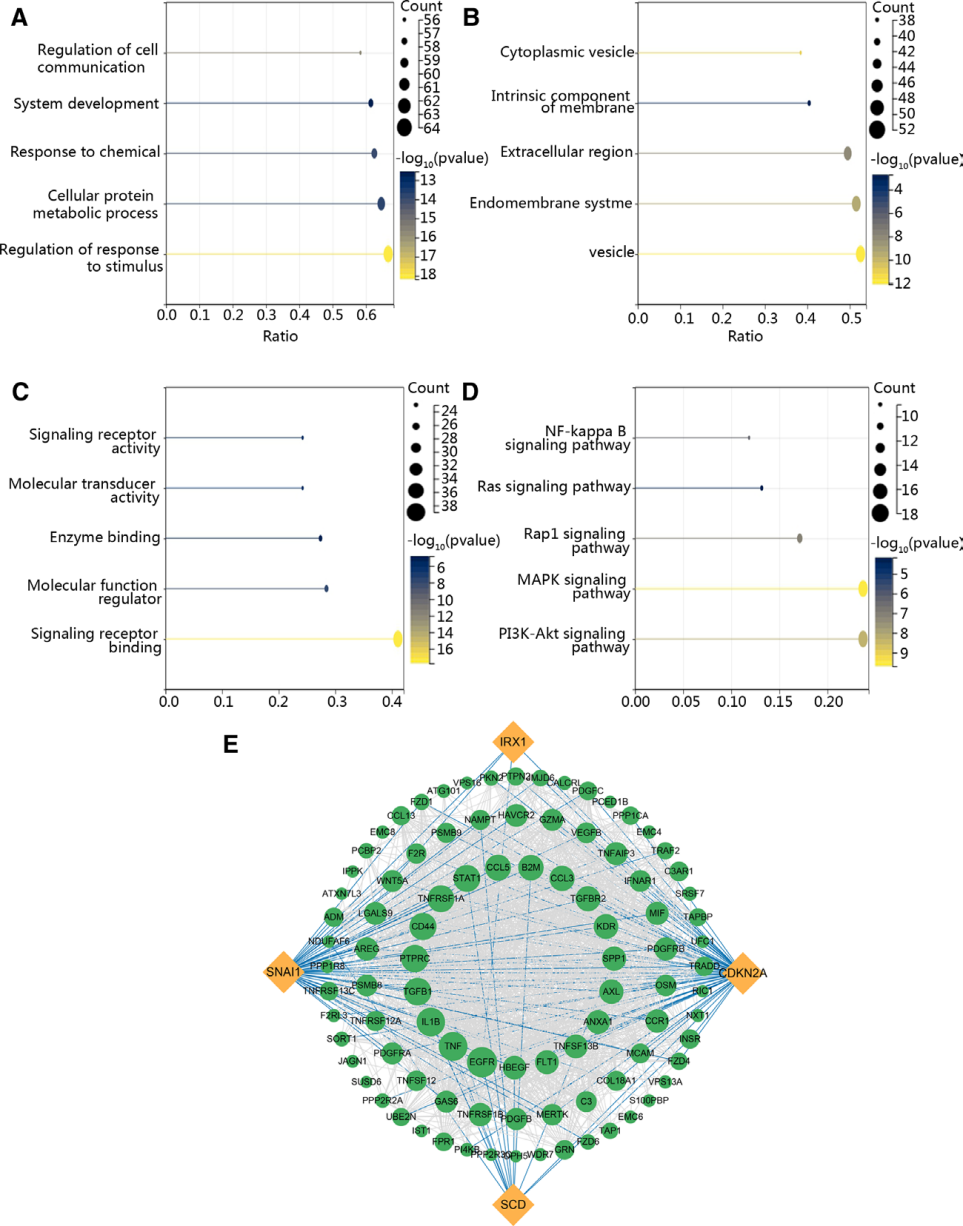
The function and interaction of hub genes, immune escape-related genes and pairs of ligand and receptor. (A) The GO enrichment analysis in the aspect of BP. (B) The GO enrichment analysis in the aspect of CC. (C) The GO enrichment analysis in the aspect of MF. (D) The KEGG enrichment analysis. (E) The PPI network among hub genes and immune escape-related genes and pairs of ligands and receptors. GO = gene ontology, KEGG = kyoto encyclopedia of genes and genomes, PPI = protein-protein interaction.

Based on the above results, we determined the enriched function of immune escape-related genes and pairs of ligands and receptors which were significantly related to the hub genes using GO and KEGG analysis. The results exhibited that these genes played vital roles in the regulation of response to stimulus, cellular protein metabolic process and response to chemical (Fig. 9A). The GO results also displayed that these genes were mainly expressed in vesicle, endomembrane system and extracellular region (Fig. 9B). In the term of MF, they were enriched in signaling receptor binding, molecular function regulator and enzyme binding (Fig. 9C). KEGG enrichment pathway included PI3K-Akt signaling pathway, MAPK signaling pathway and Rap1 signaling pathway (Fig. 9D). Additionally, we constructed a PPI network to further assess their correlation. Figure 9E exhibited that CDKN2A has the highest degree, while IRX1 has the lowest degree.

### 3.9. The drug sensitivity of hub genes

**Figure 10.**
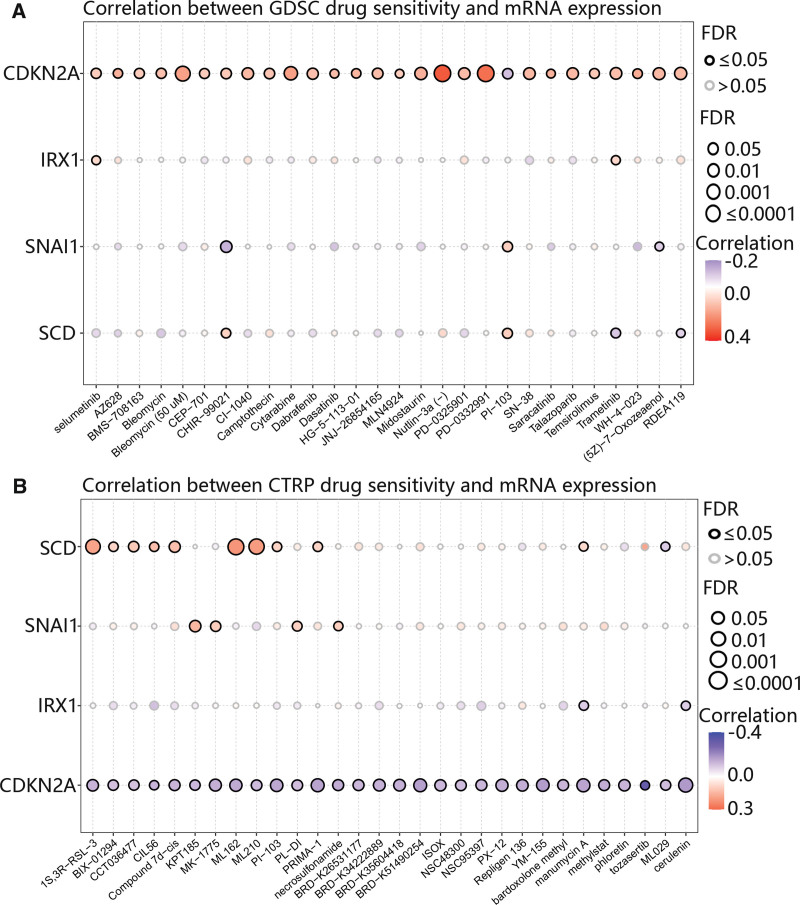
The correlation between drugs and hub genes based on GDSC and CTRP databases. (A) The correlation between drugs and hub genes in the GDSC database. (B) The correlation between drugs and hub genes in the CTRP database. CTRP = cancer therapeutics response portal, GDSC = genomics of drug sensitivity in cancer.

Finally, we assess the value of hub genes for clinical treatment using the GDSC and CTRP databases. It was obvious that CDKN2A was positively related to most drugs based on the GDSC database (Fig. 10A), In stark contrast, the CTRP database revealed a negative relationship between CDKN2A and the majority of the drugs investigated (Fig. 10B). Additionally, SCD was closely related to ML210, ML162, and 1S,3R-RSL-3 (Fig. 10B).

## 4. Discussion

PTC is a common tumor and generally has a good prognosis, but the metastasis of the tumor is usually related to a poor prognosis of PTC.^[19]^ Anoikis is a physiologically programmed apoptosis, which could maintain tissue homeostasis through eliminating detached cells.^[20]^ Moreover, anoikis has been considered as an essential factor in promoting tumor metastasis.^[21]^ Thus, anoikis may be involved in the development of PTC. Given that ARG signatures and potential mechanisms have not been studied in PTC, we identified the function of anoikis-related signatures in PTC using a comprehensive analysis of scRNA and bulk RNA.

In this study, we leveraged ARGs and the expression profiles of PTC to ascertain the differentially expressed ARGs that are closely related to the OS of PTC patients. Furthermore, a risk model based on 4 ARGs was developed with stable and effective accuracy for predicting the OS of PTC. GSVA results exhibited that the high risk group was enriched in EMT and apoptosis, which fit with previous mechanistic knowledge.

As previous studies described, the increasing level of TNFA-NFκB could enhance the suppressive function of Treg cells through inducing the proliferation of Treg cells, resulting in the immune escape of tumor cells.^[22,23]^ In addition, IL2-STAT5 could also regulate the activation of Treg cells through inducing Foxp3.^[24]^ Besides, we found that the high risk group was also enriched in angiogenesis, TNFA-NFκB signal pathway and IL2-STAT5 signal pathway. It indicated that ARGs may be involved in the development of PTC through promoting angiogenesis and inhibiting immune response. Moreover, it had been reported that CD8 T cells mediated cytotoxicity and played a vital role in immune response,^[25]^ but the activation and differentiation of CD8 T cells was negatively regulated by Treg cells.^[26]^ Furthermore, previous studies have highlighted the suppressive effects of Treg cells on CD8 + T cells, leading to increased expression of immunosuppressive molecules by tumors as a means of immune evasion.^[27]^ In our study, we observed an increased TIDE score and a decreased MSI score in the high-risk group. Moreover, the infiltration levels of CD8T cells and monocytes were downgraded, while the Treg infiltration level was upgraded. These results suggested that the high risk group was closely related to the high possibility of tumor immune escape and poor prognosis of immune checkpoint blocking therapy through activating Treg cells and inhibiting CD8 T cells.

The risk model included 4 signatures, namely CDKN2A, IRX1, SCD, and SNAI1. CDKN2A is a member of the family of cyclin-dependent kinase inhibitor genes and may be involved in the development of EMT and infiltration of immune cells through regulating LIPH expression.^[28]^ Besides, it had been widely reported that CDKN2A was related to the poor prognosis of various cancers, including hepatocellular carcinoma, gliomas and colorectal cancer.^[29–31]^ Both SCD and SNAI1 are recognized oncogenes in multiple cancer types. Liu et al found that SCD could promote the metastasis of tumor cells through mediating lipid metabolic reprogramming.^[32]^ Besides, SNAI1 have a positive effect on the development and invasion of various tumors through inducing EMT.^[33–35]^ In addition, IRX1, a member of the iroquois homeobox transcription factor family, has been identified as a tumor suppressor in several cancers, including lung adenocarcinoma, gastric cancer and invasive ductal carcinoma.^[36–39]^ In our study, we found that high expressions of CDKN2A, SCD and SNAI1 were closely related to the poor prognosis, which was consistent with previous studies. However, our results identified that IRX1 was an oncogene in PTC. It suggested that the function of IRX1 may vary depending on the type of cancer cells. Moreover, we found they were significantly related to the immune escape genes and the receptor and ligand pairs existed in the monocytes, macrophages and T cells. Combining the previous results of immune infiltration, we inferred that hub genes may activate the Tregs to inhibit the function of CD8T cells, which caused the immune escape of tumor cells and promoted the development of PTC. Moreover, while the TIDE scores and immune microenvironment analysis suggest challenges in achieving beneficial effects from immunotherapy in high-risk groups, the drug sensitivity results demonstrated that CDKN2A could be a target for chemotherapy because the low CDKN2A expression was closely related to the promising prognosis and high drug sensitivity.

However, our analysis was based on the TCGA database which was mainly containing Caucasians. Thus, the results may be not suitable for all human beings. Secondly, ensure the accuracy and reliability of our findings, it is crucial to perform large-scale clinical trials that directly validate the associations we have identified. Clinical trials would provide more comprehensive and robust evidence regarding the prognostic value of the hub genes included in our risk model. Thirdly, the results were inferred based on the bioinformation, which needs to be verified in vivo and in vitro experiments. In conclusion, we identified that high expressions of CDKN2A, IRX1, SCD and SNAI1 were closely related to the poor prognosis of PTC, and we constructed a risk model based on these hub genes with great effectiveness and stability. Moreover, we inferred that the potential mechanism of anoikis may be related to the immune escape of tumor cells through mediating the activity of Tregs cells and CD8 T cells.

## Author contributions

**Conceptualization:** Ke Zheng.

**Data curation:** Ke Zheng, Xiu-xia Zhang, Yi-fei Yang.

**Formal analysis:** Ke Zheng, Xiu-xia Zhang.

**Investigation:** Xin Yu.

**Methodology:** Ke Zheng, Xin Yu, Bin Yu.

**Supervision:** Yi-fei Yang.

**Writing – original draft:** Ke Zheng, Xiu-xia Zhang, Xin Yu, Bin Yu.

**Writing – review & editing:** Yi-fei Yang.

## Supplementary Material




