# Supplementary figures and images for: Identification and validation of a prognostic anoikis-related gene signature in papillary thyroid carcinoma by integrated analysis of single-cell and bulk RNA-sequencing
Source: Medicine (Baltimore). 2024 May 10;103(19):e38144. doi: 10.1097/MD.0000000000038144 (PMC11081552; doi:10.1097/MD.0000000000038144)

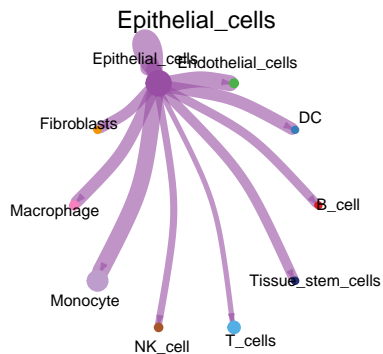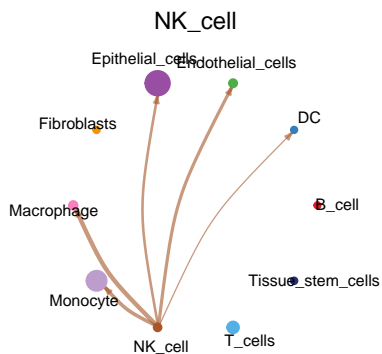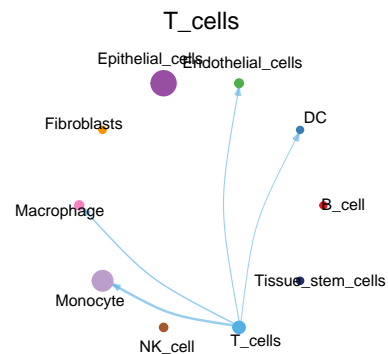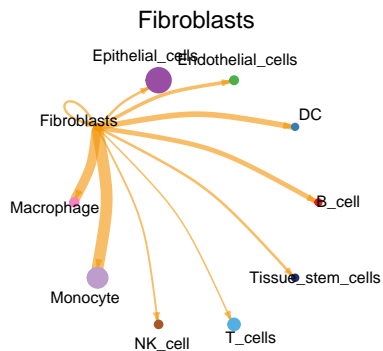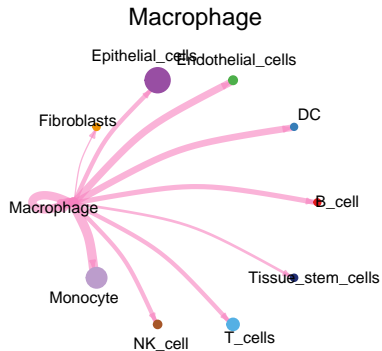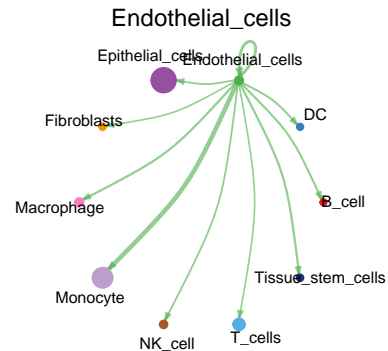

## Monocyte

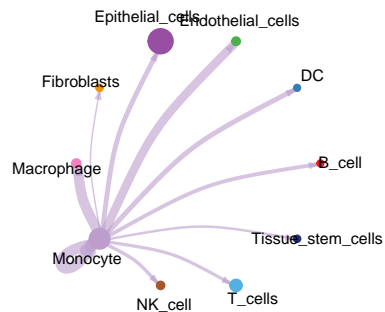

## Tissue\_stem\_cells

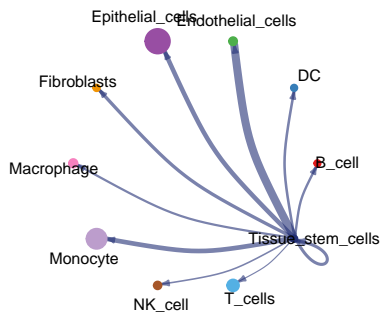

## B\_cell

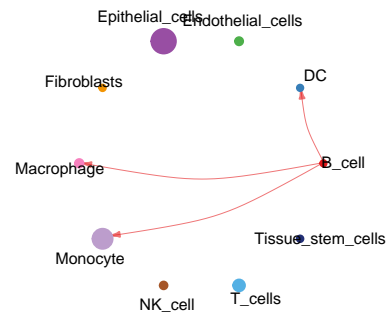

## DC

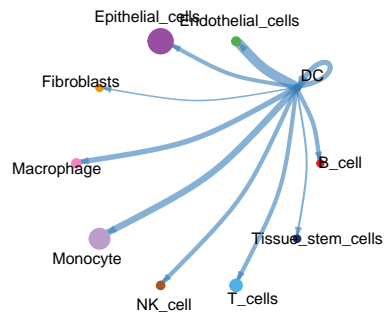

Supplement: Supplementary file 1 [file medi-103-e38144-s001.pdf]

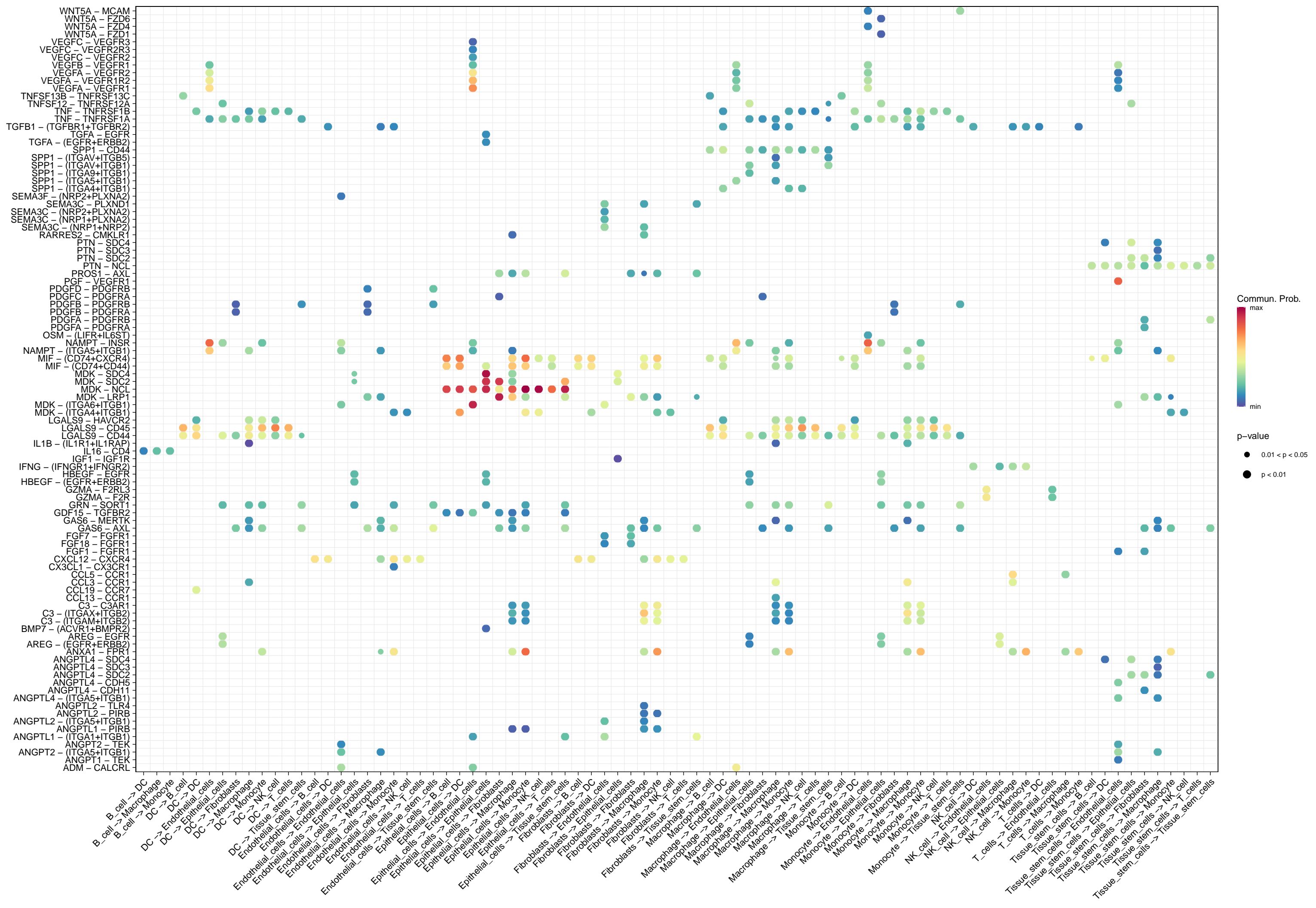

Supplement: Supplementary file 2 [file medi-103-e38144-s002.pdf]
